# Exploring Quercetin Anti-Osteoporosis Pharmacological Mechanisms with In Silico and In Vivo Models

**DOI:** 10.3390/life12070980

**Published:** 2022-06-29

**Authors:** Ying Hu, Wei Yuan, Na Cai, Kun Jia, Yunlong Meng, Fei Wang, Yurui Ge, Huiqiang Lu

**Affiliations:** 1 Ganzhou Key Laboratory for Drug Screening and Discovery, Gannan Normal University, Ganzhou 341000, China; hy939900@126.com (Y.H.); caina2022@126.com (N.C.); kunj1996@126.com (K.J.); yunlong_meng@126.com (Y.M.); w18720740019@163.com (F.W.); geyurui2021@163.com (Y.G.); 2 Jiangxi Engineering Laboratory of Zebrafish Modeling and Drug Screening for Human Diseases, Ji’an 343009, China; 3 Jiangxi Key Laboratory of Developmental Biology of Organs, Ji’an 343009, China

**Keywords:** quercetin, osteoporosis, pharmacological mechanism, network pharmacology, zebrafish

## Abstract

Since osteoporosis critically influences the lives of patients with a high incidence, effective therapeutic treatments are important. Quercetin has been well recognized as a bone-sparing agent and thus the underlying mechanisms warrant further investigation. In the current study, the network pharmacology strategy and zebrafish model were utilized to explain the potential pharmacological effects of quercetin on osteoporosis. The potential targets and related signaling pathways were explored through overlapping target prediction, protein–protein interaction network construction, and functional enrichment analysis. Furthermore, we performed docking studies to verify the specific interactions between quercetin and crucial targets. Consequently, 55 targets were related to osteoporosis disease among the 159 targets of quercetin obtained by three database sources. Thirty hub targets were filtered through the cytoNCA plugin. Additionally, the Gene Ontology functions in the top 10 respective biological processes, molecular functions, and cell components as well as the top 20 Kyoto Encyclopedia of Genes and Genomes (KEGG) pathways were depicted. The most significance difference in the KEGG pathways was the TNF signaling pathway, consisting of the Nuclear Factor Kappa B Subunit (NF-κB), Extracellular Regulated Protein Kinases (ERK) 1/2, Activator Protein 1 (AP-1), Interleukin 6 (IL6), Transcription factor AP-1 (Jun), and Phosphatidylinositol 3 Kinase (PI3K), which were probably involved in the pharmacological effects. Moreover, molecular docking studies revealed that the top three entries were Interleukin 1 Beta (IL1B), the Nuclear Factor NF-Kappa-B p65 Subunit (RelA), and the Nuclear Factor Kappa B Subunit 1 (NFKB1), respectively. Finally, these results were verified by alizarin red-stained mineralized bone in zebrafish and related qPCR experiments. The findings probably facilitate the mechanism elucidation related to quercetin anti-osteoporosis action.

## 1. Introduction

Osteoporosis (OP) is a multifactorial skeletal disease with an increased risk of fracture [1]. With the extension of mean life expectancy and aging, the prevalence of OP is progressively raising [2]. The prevalence rates of people over 50 and 60 years old were 19.2% and 32% in China according to an epidemiologic survey in 2018 [3]. The disorder of bone metabolism is highly prone to the occurrence of OP, which is characterized by a deterioration of bone mineral density, bone mass, and microstructure, and thereby easily leads to brittle fracture [4]. Approximately 300 thousand people are hospitalized owing to hip fracture, which is the most harmful type of fracture [5]. A more serious concern is that the mortality rate of patients is about 20% [5]. In light of the prediction from professionals, the number of patients with OP-related fractures will reach nearly six million by 2050 in China alone [6]. In the United States, annual fractures are projected to increase from 1.9 to 3.2 million from 2018 to 2040 [5]. OP has been attributed to the dysfunction of bone homeostasis [7]. The balance is finely modulated by osteoblasts that promote bone formation and osteoclasts that mainly absorb bone [7]. In modern medical clinics, anti-OP drugs mainly include bone resorption inhibitors, bone formation promoters, bone mineralizers, strontium ranelate, and vitamin D [8]. However, prevention and treatment, together with a diagnosis for OP, still remain under exploited thus far [9].

With the emergence of systems biology, network pharmacology has become a promising paradigm of drug development [10]. Numerous studies have shown that network pharmacology approaches can provide new insights and directions for the development of traditional Chinese medicine (TCM) resources, which is in favor of the discovery of TCM drugs [11]. Based on the network analysis of biological systems, network pharmacology provides sufficient evidence for the specific molecular mechanism between drugs and diseases [12]. Recently, many investigators have revealed that TCM drugs have great potential in the prevention and treatment of OP under high therapeutic efficacy and have low side effects [13]. Xiao et al. uncovered the treatment mechanisms of *Eucommia ulmoides* and *Epimedium brevicornu* Maxim on OP through network pharmacology analysis, where the hub genes included Mitogen-Activated Protein Kinase 1 (MAPK1), Tumor Necrosis Factor (TNF), AKT serine/threonine kinase 1 (AKT1), Cyclin D1 (CCDN1), Fos Proto-Oncogene (FOS), Jun, and Tumor Protein P53 (TP53) [9]. The active constituents and potential mechanism of Xianlinggubao in the treatment of OP has also been studied via network pharmacology methods, involving Interleukin 17 (IL-17), Hypoxia Inducible Factor 1 (HIF1), Insulin Resistance (INS), and the Th-17 signaling pathway, etc. [14]. In the aid of the network pharmacology strategy, there are also many other TCM ingredients which were explored to elucidate the treatment mechanisms of OP, such as *Sinomenii* *caulis*, *Rhizoma drynariae*, and *Astragaloside IV*, etc. [15].

Quercetin (QCT, C_15_H_10_O_7_) is a naturally occurring flavonoid which is an active ingredient in TCM preparations. This ingredient is present ubiquitously in fruits and vegetables with a variety of biological activities, such as effectively regulating cell cycle process and signals and auxin transport related to plant growth and development [16]. QCT has potential applications in anatomy, cardiovascular protection, and anti-immunotherapy [17]. Besides, anti-inflammatory and antioxidant aspects of QCT and its derivatives have been well recognized [18]. The recent network pharmacology study of QCT showed that the therapeutic effect on oral lichen planus was modulated by the inflammatory factor IL6 [19]. In terms of bone homeostasis, QCT could promote new local bone formations and could be applied to a bone-graft material [20]. QCT upregulated the alkaline phosphatase activity of osteoblastic MC3T3-E1 cells and improved new bone regeneration in localized bone defects in vivo [21]. QCT exhibited a protective effect on bone homeostasis to a large extent despite negative results from a few studies [22]. Additionally, QCT regulated bone metabolism through the MAPK signaling pathway and facilitated osteoclast activation by regulating the RANKL (Receptor Activator of Nuclear Factor-κB Ligand)/RANK/OPG (Osteoprotegerin) signal pathway [23]. However, despite these advances, the pharmacological mechanism of QCT on anti-OP remains limited.

Zebrafish have a high homology with human genes, which can be used to establish an osteoporosis model [24]. Herein, we focused on the elucidation of the mechanism of QCT on anti-OP via network pharmacology and molecular docking methods in the present study. We obtained the structure, oral bioavailability (OB), and drug-likeness (DL) of QCT from the Traditional Chinese Medicine Systems Pharmacology Database and Analysis Platform (TCMSP). The potential molecular targets of QCT were identified from the TCMSP, Swiss target prediction, and PubChem. We next screened OP-related targets in GeneCards, DisGeNET, the Online Mendelian Inheritance in Man (OMIM), and the Therapeutic Target Database (TTD). Subsequently, the intersection in the Venn diagram was recognized as the potential targets of QCT against OP. These targets were selected to construct a protein–protein interaction (PPI) network through the STRING database. The 30 key targets were selected based on the degree centrality (DC), betweenness centrality (BC), closeness centrality (CC), and subgraph centrality (SC). A Gene Ontology (GO) and Kyoto Encyclopedia of Genes and Genomes (KEGG) terms enrichment analysis were performed on the hub targets. Furthermore, the most significance difference in the KEGG analysis was indicated. The target-pathway network was depicted by Cytoscape software and the result of the network revealed the core targets. Docking studies for QCT were then carried out to identify the strongest binding affinity among the core targets. Finally, the zebrafish osteoporosis model was induced by glucocorticoid dexamethasone sodium phosphate, and the rescue experiment was carried out by QCT. Alizarin red staining was used to quantify the degree of skull mineralization. Additionally, a quantitative real-time PCR-associated estimation of the molecular docking results was performed.

## 2. Results

### 2.1. Screening of Potential Targets

The OB and DL of QCT were 46.43% and 0.28, respectively. OB refers to the amount of medication that actually enters the body circulation after oral absorption; DL refers to the similarity of the compounds with known drugs [25]. Both of the parameters are the two most important indicators for evaluating pharmacokinetic characteristics via bioinformatics [25]. It is clear that the estimation values of the OB and DL were higher than 20% and 0.18, which are the most common thresholds, respectively [25]. Hence, the QCT had a good drug likeness because of satisfying the OB and DL index [26].

**Figure 1:**
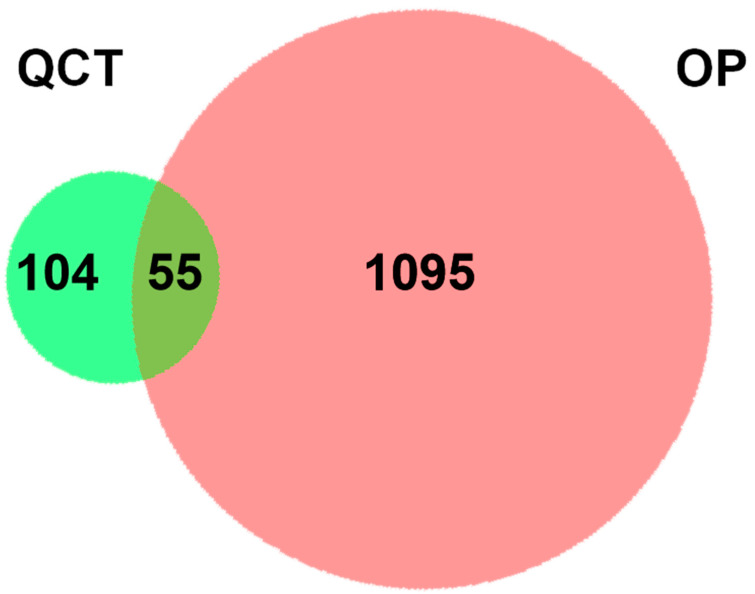
A two-set Venn diagram illustrating the relationship between QCT and OP-related targets. Light green and red represent QCT and OP-related targets, respectively. The intersection depicts a total of 55 potential pharmacological targets for QCT in the treatment of OP.

After the query and analysis of the TCMSP, Swiss Target Prediction, and PubChem database websites, a total of 159 QCT-related targets were obtained after integration and deduplication (Table S1). We also collected 1150 targets associated with OP from the GeneCards (relevance score ≥ 10), DisGeNET (score-gda ≥ 0.1), OMIM, and TTD database (Table S2) [27]. The above targets from QCT and OP were analyzed by a Venn diagram generated by an online platform (Figure 1). As a result, 55 common targets were observed, revealing the correlations with OP and QCT (Table S3).

### 2.2. PPI Network Construction and Hub Target Analysis

**Figure 2:**
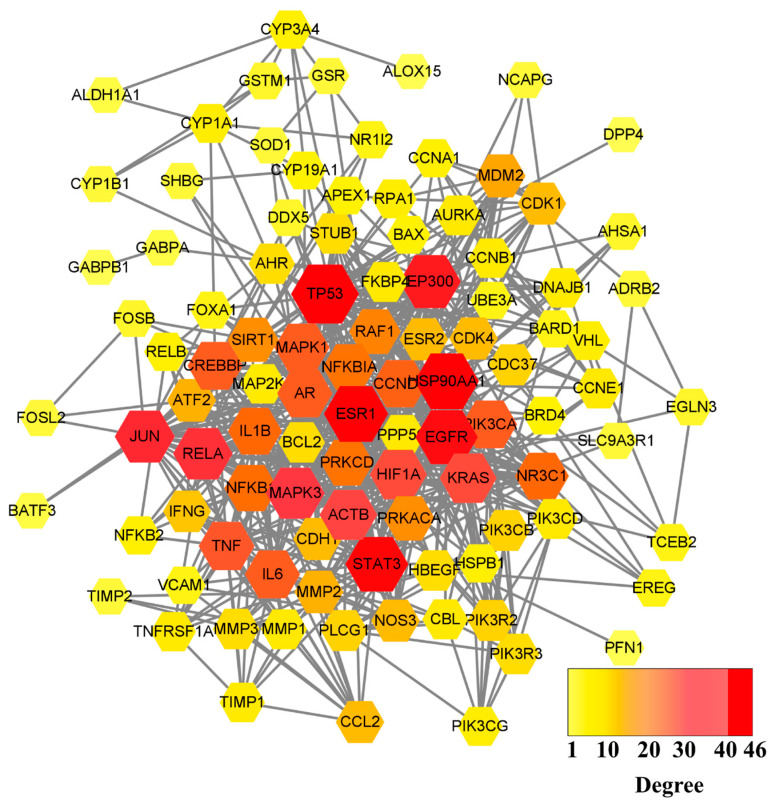
PPI network graph of potential targets. The node size is proportional to its degree value where the bigger size means the larger degree value.

After the 55 common targets were submitted to the STRING database, PPI information was obtained. The network graph comprised 100 nodes and 656 edges and was depicted by Cytoscape software (Figure 2). After screening, there were 30 intersection targets whose values of four evaluation criteria (DC, BC, CC, and SC) were greater than the median [28]. These genes were considered key targets (Table 1). As the characteristic parameter, the DC value was used to determine the significance of the putative proteins. The degree of the top ten potential targets associated with OP and QCT were Beta-actin (ACTB), TP53, IL6, TNF, Estrogen Receptor 1 (ESR1), Jun, Epidermal Growth Factor Receptor (EGFR), IL1B, Mitogen-Activated Protein Kinase 3 (MAPK3), and Hypoxia Inducible Factor-1 Subunit Alpha (HIF1A).

### 2.3. GO and KEGG Pathway Enrichment Analyses

**Figure 3:**
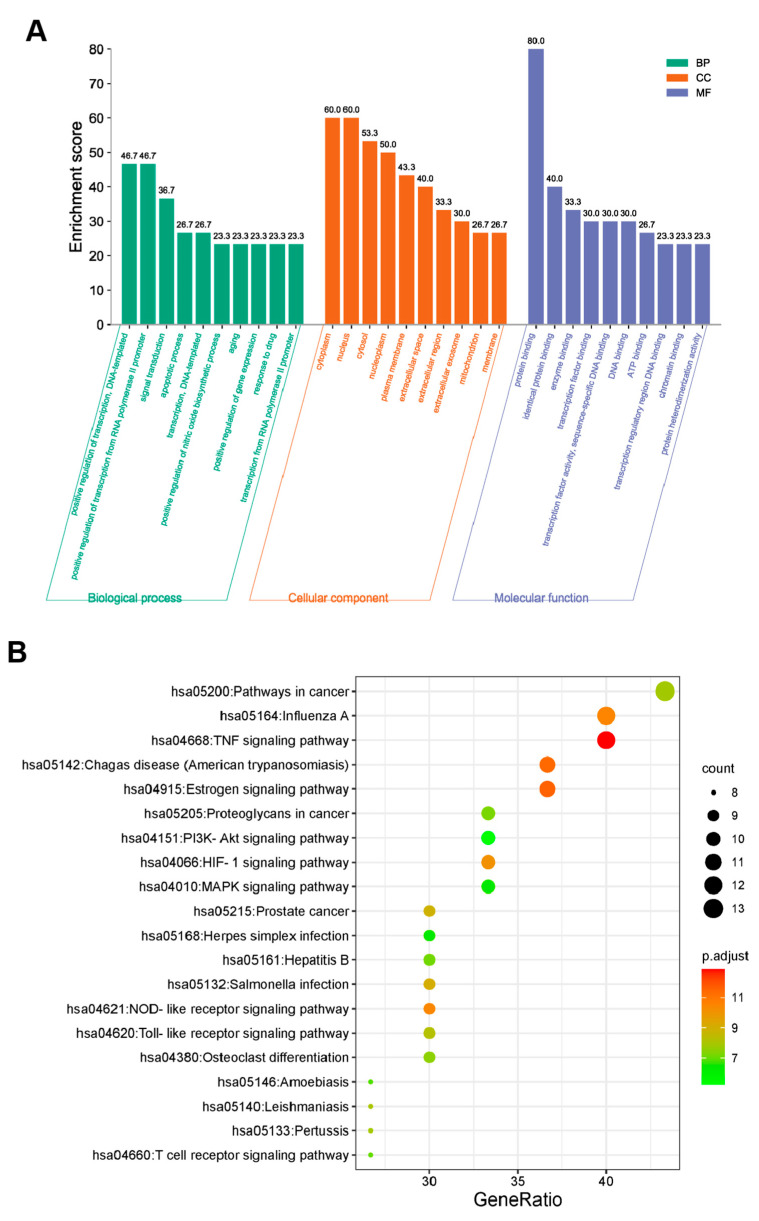
GO terms and KEGG pathway enrichment analysis of 30 common targets (*p*-value < 0.05). (**A**) GO enrichment analysis of the intersection targets. The abscissa represents the GO term; the ordinate represents the proportion of genes. Green, orange, and blue represent the top 10 biological process, cellular components, and molecular functions, respectively. (**B**) The top 20 significant KEGG pathways. The abscissa depicts the proportion of genes, while the ordinate indicates the name of the KEGG signal pathway. The dot size and color scales indicated the number of enriched genes and the different thresholds for the *p*-value.

In terms of the 30 key targets, GO and KEGG pathway enrichment analyses were initiated based on the DAVID database. Specifically, a total of 255 GO terms were retrieved and annotated, which contained 186 biological processes (BP), 48 molecular functions (MF), and 21 cell components (CC). All of the top 10 key targets were graphed using the bioinformatics online platform (Figure 3A). The BP mainly involved the positive regulation of DNA-templated transcription, the positive regulation of transcription from the RNA polymerase II promoter, the signal transduction, the apoptotic process; the DNA-templated transcription, the positive regulation of the nitric oxide biosynthetic process, the aging, the positive regulation of the gene expression, the response to the drug, and transcription from the RNA polymerase II promoter. The MF was mainly related to protein binding, identical protein binding, enzyme binding, transcription factor binding, transcription factor activity, sequence-specific DNA binding, DNA binding, ATP binding, transcription regulatory region DNA binding, chromatin binding, and protein heterodimerization activity. Additionally, the CC was mainly linked to the cytoplasm, nucleus, cytosol, nucleoplasm, plasma membrane, extracellular space, extracellular region, extracellular exosome, mitochondrion, and membrane.

Additionally, we filtered the KEGG pathway according to the *p*-value (≤0.05) to elucidate the critical pathways among the 55 potential targets in terms of OP therapy [29,30]. Ultimately, 96 significantly different signaling pathways were gained (Table S4). The top 20 pathways included the pathways in cancer (hsa05200), Influenza A (hsa05164), the TNF signaling pathway (hsa04668), Chagas disease (American trypanosomiasis) (hsa05142), the Estrogen signaling pathway (hsa04915), Proteoglycans in cancer (hsa05205), the PI3K-Akt signaling pathway (hsa04151), the HIF-1 signaling pathway (hsa04066), the MAPK signaling pathway (hsa04010), prostate cancer (hsa05215), herpes simplex infection (hsa05168), Hepatitis B (hsa05161), salmonella infection (hsa05132), the NOD-like receptor signaling pathway (hsa04621), the toll-like receptor signaling pathway (hsa04620), osteoclast differentiation (hsa04380), Amoebiasis (hsa05146), Leishmaniasis (hsa05140), Pertussis (hsa05133), and the T cell receptor signaling pathway (hsa04660), which were selected to form a bubble chart using a bioinformatics online platform (Figure 3B).

**Figure 4:**
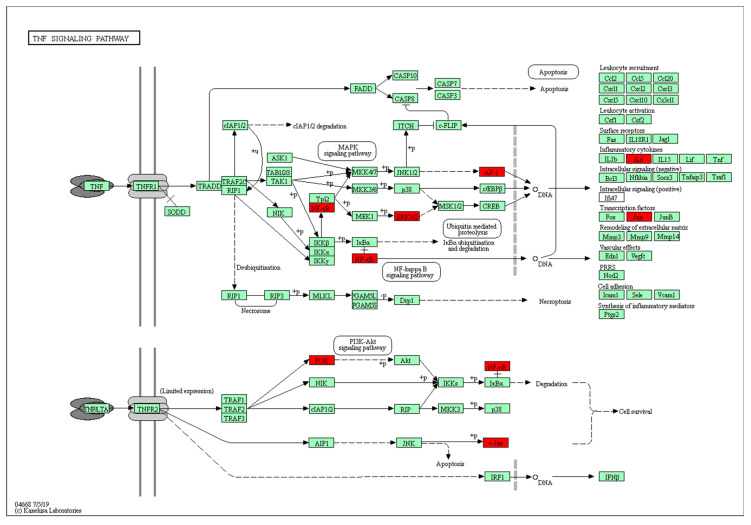
Potential hub targets of QCT toward anti-OP activity in the TNF signaling pathway generated by the KEGG mapper. The red rectangles indicate the identified proteins and the green rectangles indicate the unidentified proteins.

Among the KEGG-enriched pathways, the TNF signaling pathways had the most significance difference. The predicted targets in the TNF signaling pathway are shown in Figure 4. In the TNF signaling pathway, the targets marked in red were deemed to play a role in the mechanism of QCT against OP. They were NF-κB, ERK1/2, AP-1, IL6, Jun, and PI3K, respectively.

**Figure 5:**
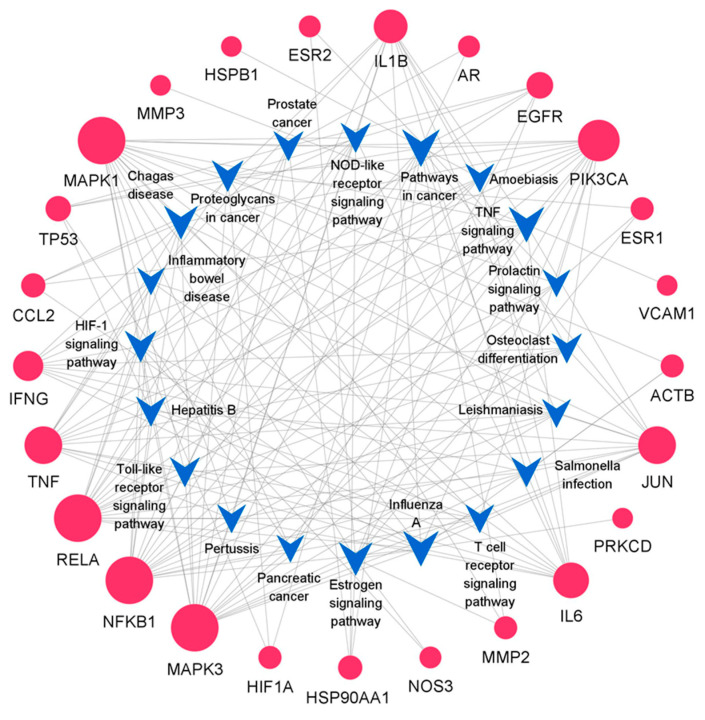
Target-pathway network. The blue V-shaped patterns and red circles represent different pathways and targets. The size of the circle indicates the degree of importance in the pathway.

The results of the target-pathway network indicated that nine genes appeared frequently (≥10 times) in the top 20 KEGG pathways (Figure 5), which suggested that nine genes were crucial for the pharmacological mechanism [31]. The nine core proteins were RelA, NFKB1, MAPK1, MAPK3, the PI3-Kinase P110 Subunit Alpha (PIK3CA), TNF, Jun, IL6, and IL1B, respectively.

### 2.4. Molecular Docking

Through a molecular docking analysis, the binding and interaction between the target proteins and the small molecules could be predicted. The molecular docking of QCT with nine important targets IL1B, RelA, NFKB1, MAPK1, TNF, IL6, Jun, MAPK3, and PIK3CA were studied. These targets were selected because they both represented the key nodes of the PPI network and played significant roles in the KEGG signaling pathways. The molecular docking results are shown in Table 2, where the lower the affinity (kcal/mol) is, the greater the binding force is predicted.

We selected the top three genes with a high affinity and then drew a 3D docking map with PyMOL and a 2D diagram with LigPlus+. The docking results suggested that the receptor–ligand interactions of the QCT and OP were involved in hydrophobic interactions and polar interactions. The details are described below.

**Figure 6:**
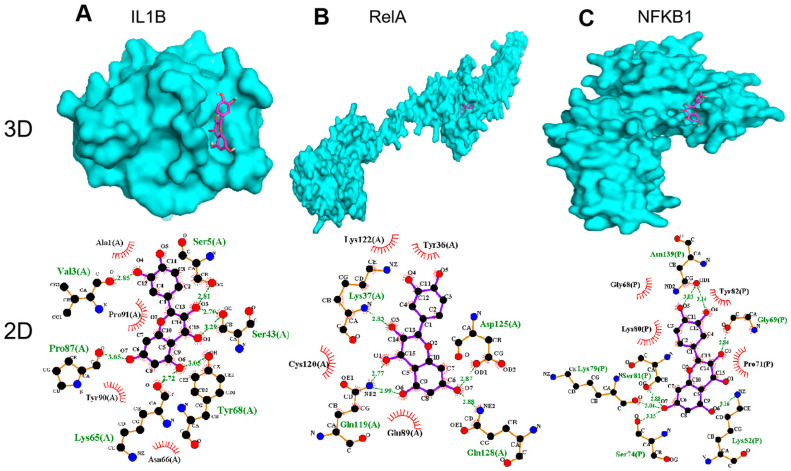
Molecular models of QCT binding to its predicted protein targets: (**A**) IL1B, (**B**) RelA, and (**C**) NFKB1 (shown as 3D and 2D diagrams).

In Figure 6A, QCT is bound to the active pocket of IL1B. Their interaction is formed by a stable hydrophobic core including several nonpolar residues in IL1B (Ala1, Pro91, Tyr90, and Asn66). Additionally, the hydroxyl groups of Val3 (2.85 Å), Ser5 (2.81 Å), Ser43 (3.29 Å), Pro87 (3.05 Å), Lys65 (2.72 Å), and Tyr68 (3.05 Å) within the main chains contact QCT through hydrogen bonds, which makes the interaction of the whole structure more stable.

In addition, the results in Figure 6B show that QCT was bound to the active pocket of RelA by several hydrophobic interactions with the surrounding residues in RelA (Lys122, Tyr36, Cys120, and Glu89). Additionally, QCT formed five H-bonds with Lys37 (2.83 Å), Asp125 (2.87 Å), Gln128 (2.88 Å), Gln119 (2.77 Å), and Gln119(2.99 Å).

As displayed by Figure 6C, QCT was prominently observed to interact with the hydrophobic groups of four residues in NFKB1 (Gly68, Lys80, Tyr82, and Pro71). Furthermore, seven hydrogen bonds from Lys52 (3.16 Å), Gly69 (2.84 Å), Ser81 (2.88 Å), Lys79 (3.04 Å), Ser74 (3.15 Å), Asn139 (3.03 Å), and Asn139 (3.14 Å) further enhanced the interaction between QCT and the NFKB1 protein.

### 2.5. Anti-Osteoporosis Effect in Zebrafish

**Figure 7:**
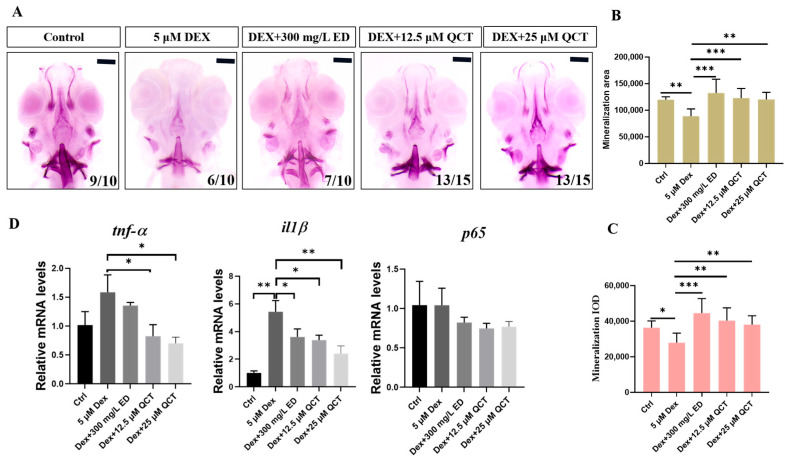
The effects of QCT on osteoporosis in zebrafish model. (**A**) Images of the dorsal aspect head bone stained with alizarin red staining in 9-dpf zebrafish. (**B**) The effect of QCT on bone mineralization area. (**C**) The effect of QCT on cumulative optical density. (**D**) Relative mRNA expression levels in zebrafish. Data are expressed as means ± SD, * *p* < 0.05, ** *p* < 0.01, *** *p* < 0.001.

To detect whether QCT has effects on anti-osteoporosis, dexamethasone (DEX), a kind of glucocorticoid, was used to construct a zebrafish model [32]. We used alizarin red staining to investigate the effects of bone formation (Figure 7A). Compared with the control group, the bone mineralization area (BMA) and cumulative optical density (IOD) had a significant decrease in the DEX-induced OP model (*p* < 0.05). Compared with the model group, the BMA and IOD of the zebrafish skulls in the positive control Etidronate disodium (ED) and QCT groups were significantly increased at 12.5 and 25 µM (Figure 7B,C). In order to verify the hypothetical targets, several molecular docking gene targets were selected for qRT-PCR analysis. The relative mRNA expression levels of *tnf-α* (*p* < 0.5), *il1β* (*p* < 0.01), and *p65* increased in the DEX group while they reduced in the ED and QCT groups (Figure 7D). These results indicated that QCT had an anti-osteoporosis activity in vivo.

## 3. Discussion

With the rapid growth of the global population and the increasingly serious problem of population aging, the OP disease is a growing health problem worldwide [33]. Hence, a large number of related research has emerged to meet the challenge, but thus far, prevention and treatment, together with the diagnosis for OP, still remains inadequate [9]. The bone protective effects of QCT has been widely established [34]. Here, network pharmacology and molecular docking approaches were used to uncover the potential mechanism of QCT in the treatment of OP in this study. Additionally, we employed the DEX-induced OP model of zebrafish larvae to verify the effect and mechanism of QCT.

Firstly, 55 common targets screening for QCT and OP were illustrated in the Venn diagram. In the PPI network, 30 key targets were picked out according to four correlations (DC, BC, CC, and SC). The results of the GO Enrichment analysis suggested that the mechanism of action was mainly involved in 186 biological processes (BP), 48 molecular functions (MF), and 21 cell components (CC). In terms of the KEGG enrichment analysis, the pathways in cancer (hsa05200), Influenza A (hsa05164), the TNF signaling pathway (hsa04668), Chagas disease (American trypanosomiasis) (hsa05142), the Estrogen signaling pathway (hsa04915), etc., were the vital signaling pathways linked to anti-OP. The targets (Jun, HSP90AA1, MMP2, HIF1A, EGFR, RelA, NFKB1, AR, IL6, PIK3CA, MAPK1, TP53, and MAPK3) were enriched in the pathways in cancer. A therapeutic mechanism for the pathways in cancer has been observed for other active TCM molecules, such as the Xianlinggubao capsule, resveratrol, and Wuling Powder [35]. The influenza A (hsa05164) pathway ranks the second in the KEGG annotations, including the IL6, Jun, IFNG, PIK3CA, IL1B, MAPK1, CCL2, TNF, RelA, NFKB1, ACTB, and MAPK3 targets. The roles of the TNF signaling pathway, Chagas disease, and the Estrogen signaling pathway were also frequently reported in anti-OP activities [36].

Among the KEGG-enriched pathways, the TNF signaling pathways had the most significance difference (*p*-value). TNF had an important effect on bone metabolism which is consistent with that previously reported in the Zhao’s study [37]. Therefore, we further visually analyzed the TNF signaling pathway using the KEGG mapper. Six targets (NF-κB, AP-1, ERK1/2, PI3K, IL6, and Jun) in the TNF signaling pathways were speculated to involve the molecular mechanism with therapeutic relevance. The transcription factors NF-κB and AP-1 were capable of activating the TNF signaling pathways. The NF-κB signaling pathway, including RelA/p65, is a key regulator of bone remodeling [38]. Transcription factor AP-1 can regulate the neurite growth of human bone marrow mesenchymal stem cells induced by laminin-1 [39]. ERK1/2 is also an important kinase for bone homeostasis [40]. For instance, the bone protection from artemisinin treatment may be mediated by the upregulated ERK1/2 pathway in glucocorticoid-induced insults on primary cultured rat BMSCs [41]. Additionally, three targets (PI3K, IL6, and Jun) associated with anti-OP have been reported previously [42].

Furthermore, nine of the thirty key targets (IL1B, RelA, NFKB1, MAPK1, TNF, IL6, Jun, MAPK3, and PIK3CA) were frequently (>10 times) present in the top 20 KEGG pathway analyses. To further explore the underlying mechanisms of the effects, we performed a molecular docking of the nine targets. The docking scores of QCT and the frequently (>10 times) present genes were found to be negative, indicating that they could bind spontaneously. The top three goals for the docking scores were IL1B, RelA, and NFKB1, respectively, since a score (less than −5 kcal/mol) indicated a strong binding activity [42]. These targets have been shown to play important roles in anti-OP according to the literature [43]. In terms of IL1B, the inhibition of this target could lead to a reduction in osteoclasts and osteoblastogenesis, thereby affecting bone resorption and bone formation [44]. NF-κB proteins were implicated to take a critical part in bone development [45]. Additionally, NF-κB-mediated inflammation could regulate the key signaling pathways for osteogenesis [46]. As for RelA/p65, it can block the RANKL-induced JNK-BID pathway, thereby accelerating osteoclasts divergence [47]. Additionally, the NFKB1 protein is related with the rapid reduction in bone mass [48]. Therefore, the IL1B and NF-κB (RelA and NFKB1) signaling pathways are key targets to treat osteoporosis phenotypes. Intriguingly, RelA and NFKB1 were enriched in the TNF pathway, thus further underscoring the importance of TNF.

Zebrafish could be used to construct an osteoporosis model because of their transparent body and bone structure, their metabolism, as well as their signal pathway which is highly similar to human beings [49]. In this study, DEX was employed to construct the osteoporosis model. The results of alizarin red staining showed that the BMA and IOD significantly increased in the QCT group compared with the DEX model group. The relative mRNA levels of *il1β* (*p* < 0.01), *tnf-α* (*p* < 0.5), and *RelA*/*p65* decreased compared with the DEX model group, which was consistent with the network analysis results.

In short, the potential targets of the pharmacological mechanism of QCT on anti-OP activity have been uncovered with in silico and in vivo models. However, the present study highly relies on network pharmacological analysis to predict the potential intersection targets of QCT and OP. In fact, the targets from websites are not comprehensive owing to the limited literature. Hence, many targets that probably exhibit crucial effects have not yet been predicted by network analysis. In further research, this limitation can be well resolved through multi-omics integration with in vivo models. Taken together, the presented findings indicated the potential anti-OP mechanism of QCT, which may provide a broad direction for further studies on the exact mechanism of QCT in the treatment of OP. However, the exact regulation mechanism still needs to be further verified in mammalian models in consideration of further clinical applications.

## 4. Materials and Methods

### 4.1. Prediction of Target Genes Associated with OP

We screened OP-related targets from GeneCards (https://www.genecards.org (accessed on 27 October 2021)), DisGeNET (http://www.disgenet.org (accessed on 10 February 2021)), OMIM (https://www.omim.org (accessed on 29 September 2021)), and TTD (http://db.idrblab.net/ttd/ (accessed on 29 September 2021)). GeneCards is a well-established, searchable, and integrative database that offers concise genomic-related information on annotated and predicted human genes [50]. DisGeNET is a comprehensive platform including a wealth of gene-disease association datasets [51]. OMIM is an online catalog of heritable or hereditary genetic diseases in humans [52]. TTD provides abundant therapeutic target resources containing information relating to the interactions between drugs and targets as well as targets and diseases [53].

### 4.2. Acquisition of QCT Structure and Target Information

Briefly, we gained the structure and target information of QCT from the TCMSP (https://tcmsp-e.com/ (accessed on 20 November 2013)) using QCT as the search term. The TCMSP is a system pharmacology platform focusing on Chinese herbal medicines that records the relationships between drugs, targets, and diseases [54]. The 3D structure of QCT was acquired from the PubChem website (https://pubchem.ncbi.nlm.nih.gov/ (accessed on 9 August 2021)), and the geometry of QCT was optimized in Avogadro (Version 1.0.2) with the MM94 force field. The optimized 3D structure was imported into the TCMSP, PubChem, and Swiss target prediction (http://www.swisstargetprediction.ch/ (accessed on 20 July 2019)) to achieve the QCT-related targets.

### 4.3. Construction of PPI Network for Intersection Target of OP and QCT

The QCT-related and OP-related targets were uploaded to the website to draw the Venn diagram (http://www.biovenn.nl/index.php (accessed on 20 October 2008)) [55]. The intersection elements of the Venn diagram were the candidate targets of QCT in the treatment of OP. To illustrate the interactions between the QCT-related and OP-related targets, a PPI network was parsed from the STRING database (http://string-db.org (accessed on 35 August 2021)). “*Homo sapiens*” was selected as the parameter in the column of species. The minimum interaction score required was higher than 0.7; meanwhile, the max number of interactors was no more than 50, under hiding the disconnected nodes. The PPI was subjected to a network topological analysis by Cytoscape 3.8.2 software [56]. The plug-in cytoNCA of Cytoscape was used to obtain the core targets, and the screening conditions included DC, BC, CC, and SC. DC is the most direct metric to characterize node centrality in network analysis [57]. The greater degree of a node means that the node has more centrality, and that the node is more important in the network [57]. BC, a measure of node centrality, is usually calculated as a score of the shortest path between the pairs of nodes that pass through the node of interest [57]. CC refers to the measurement of the average distance from one node to others [57]. SC characterizes a centrality algorithm consisting of all the participation in the network [57].

### 4.4. GO and KEGG Pathway Enrichment Analyses

The functional annotation of the core targets were performed using the DAVID database (https://david.ncifcrf.gov (accessed on 15 December 2021)) for the GO functions and KEGG pathways enrichment analysis [58]. The *p*-value ≤ 0.05 was designated as the threshold value for statistical significance. A GO analysis could further explain the intersection genes in the molecular function, biological processes, and cellular composition of anti-OP processes. The KEGG enrichment analysis revealed the major signaling pathways involved in anti-OP for QCT. The top 10 GO terms and 20 KEGG pathways were visualized through column and bubble charts, respectively, using the online platform WeiShengxin (http://www.bioinformatics.com.cn/ (accessed on 10 October 2021)). Additionally, a target-pathway network was described by Cytoscape software. Potential hub targets of QCT toward anti-OP activity in the TNF signaling pathway were constructed using the KEGG mapper.

### 4.5. Molecular Docking

Among the results of the KEGG pathway analysis, key targets which appeared frequently (>10 times) were docked with QCT. The crystal structures of the targets were obtained from the PDB protein database (http://www.pdb.org/ (accessed on 10 October 2021)). The docking pockets were predicted through the DoGSiteScorer server (https://proteins.plus/ (accessed on 23 November 2021)) [59]. The pre-docking procedures were implemented on AutoDock Tools (ADT 1.5.6), which contained five steps, namely, separating ligands, removing water, adding non-polar hydrogen, calculating the Gasteiger charge, and saving as a pdbqt file [60]. Subsequently, the grid box was setted, and the center was located at the center of the active site. The spacing parameter of the box was defined as 0.375 by default; meanwhile, a grid of 60*60*60 points in the X, Y, and Z directions was built. Eventually, molecular dockings were accomplished through AutoDock Vina, and the complexes of the proteins and compounds were visualized by PyMOL software v.1.7.1.0 [61]. Additionally, the 2D diagrams for the active site interactions were displayed by LigPlot+ v.2.2.4 [62].

### 4.6. Experimental Verification

#### 4.6.1. Osteoporosis Model

The wild type zebrafish were cultured in an E3 embryo culture medium supplied with a 0.003% 1-phenyl-2-thiourea (PTU) solution at 28 °C. These embryos at 5 dpf were placed into a six-well plate, each of who’s well contained 20 larvae. The zebrafish larvae were divided into four groups including the control group, model group, model + positive rescue group, and model + compound group. The control group was maintained in the medium with a working-PTU solution. The model group was treated with 5 μM DEX. The model + positive rescue group and model + compound group were exposed to DEX and 300 mg/L ED, DEX and 12.5 μM QCT, and DEX and 25 μM QCT, respectively. The processing time was from 5 to 9 days.

#### 4.6.2. Alizarin Red Staining

At 9 dpf, all the zebrafish larvae were anesthetized by 0.02% tricaine. After the removal of the tricaine solution, the zebrafish were fixed in 4% paraformaldehyde at 4 °C overnight and were transferred into a bleach solution (3% H_2_O_2_, 0.5% KOH) for 1 h. Next, the larvae were washed with a Polybutylece Terephthalate (PBT) solution for 5 min, which was repeated 3 times. A gradient elution was immediately carried out with 25% methanol-75% PBT, 50% methanol-50% PBT, and 100% methanol for 5 min each time, respectively. Subsequently, the zebrafish were stored at—20 °C overnight with 20% DMSO-80% methanol. In the next day, the samples were rehydrated with a PBT/methanol series (0%, 25%, 50%, 75%, and 100%). Then, all the samples were stained with alizarin red at room temperature in the dark for more than 12 h. Finally, the zebrafish were washed with PBT until colorless and were stored with glycerin. The dorsal aspect of the zebrafish head was photographed using a fluorescence stereomicroscope (Leica M205 FA stereomicroscope). The staining area and IOD were calculated by Image-ProPlus 6.0 software to investigate the bone mineralization area and the bone mineral density.

#### 4.6.3. Quantitative Real-Time PCR Analysis

The total RNA of the zebrafish was extracted from different groups of larvae (n = 40 larvae/group) using TransZol Up reagent (ET111–01). Then, the RNA was reverse transcribed into cDNA through a reverse transcription kit (EasyScript^®^ One-Step gDNA Removal and cDNA Synthesis SuperMix, AE311–02). The mRNA expression levels were detected by a real-time fluorescence quantitative PCR kit (TransStart^®^ Tip Green qPCR SuperMix, AQ141–02). The results were normalized to β-actin expression and quantified by the 2^−ΔΔCt^ method. Forward and reverse primers were listed in Table S5.

#### 4.6.4. Statistical Analysis

GraphPad Prism Version 8.00 was used for statistical analysis. All data were expressed as means ± SD, and a *p*-value less than 0.05 was considered to be statistically significant.

## 5. Conclusions

In this study, the network pharmacology approach and in vivo models were carried out to investigate the mechanism of QCT against OP. As a result, 55 potential targets were presumably associated with the action. The KEGG pathway enrichment results indicated that the TNF signaling pathway played an important role in the pharmacological effects. Additionally, targets that appeared frequently (>10 times) (IL1B, RelA, NFKB1, MAPK1, TNF, IL6, Jun, MAPK3, and PIK3CA) were identified. Moreover, molecular docking studies revealed that the targets (IL1B, RelA, and NFKB1) displayed the top three strongest binding affinity, which probably explained the anti-OP activity of QCT. Furthermore, the results of the zebrafish model and the qPCR experiments were also consistent with the action. Taken together, the presented findings systematically analyzed the potential mechanism of QCT in the treatment of OP, which may provide a broad direction for further studies on the exact mechanism of QCT in the treatment of OP.

## Figures and Tables

**Table 1:** Key targets prediction of the anti-OP effect of QCT.

No.	Symbol ID	Protein Name
1	ACTB	Beta-actin
2	TP53	Tumor Protein P53
3	IL6	Interleukin 6
4	TNF	Tumour Necrosis Factor
5	ESR1	Estrogen Receptor 1
6	JUN	Jun Proto-Oncogene, AP-1 Transcription Factor Subunit
7	EGFR	Epidermal Growth Factor Receptor
8	IL1B	Interleukin 1 Beta
9	MAPK3	Mitogen-Activated Protein Kinase 3
10	HIF1A	Hypoxia Inducible Factor-1A
11	HSP90AA1	Heat Shock Protein 90 Alpha Family Class A Member 1
12	MAPK1	Mitogen-Activated Protein Kinase 1
13	NOS3	Nitric Oxide Synthase 3
14	IFNG	Active Interferon Gamma
15	AR	Androgen Receptor
16	RelA	NF-Kappa-B Transcription Factor p65
17	CCL2	Monocyte Chemoattractant Protein-1
18	ESR2	Estrogen Receptor Beta
19	AHR	Aromatic Hydrocarbon Receptor
20	VCAM1	Vascular Cell Adhesion Protein 1
21	MMP2	Matrix Metalloproteinase-2
22	NFKB1	Nuclear Factor NF-Kappa-B P105 Subunit
23	PIK3CA	PI3-Kinase P110 Subunit Alpha
24	CYP3A4	Cytochrome P450 Family 3 Subfamily A Member 4
25	CYP1A1	Cytochrome P450 Family 1 Subfamily A Member 1
26	PRKCD	Protein Kinase C Delta
27	HSPB1	Heat Shock Protein Family B (Small) Member 1
28	MMP3	Matrix Metallopeptidase 3
29	CYP19A1	Cytochrome P450 Family 19 Subfamily A Member 1
30	SOD1	Superoxide Dismutase 1

**Table 2:** Docking scores of QCT with potential targets.

Targts	PDB ID	Affinity (kcal/mol)
IL1B	9ILB	−7.1
RelA	1NFI	−6.9
NFKB1	1SVC	−6.8
MAPK1	6G54	−6.6
TNF	1TNR	−6.5
IL6	4O9H	−6.3
Jun	6Y3V	−6.3
MAPK3	2ZOQ	−5.1
PIK3CA	6OAC	−4.7

## Supplementary Materials

The following supporting information can be downloaded at: https://www.mdpi.com/article/10.3390/life12070980/s1, Table S1: predicted targets of quercetin, Table S2: targets associated with osteoporosis, Table S3: putative therapeutic targets of QCT mediating its effects against OP, Table S4: the results of the GO and KEGG pathway enrichment, and Table S5: forward and reverse primers.

## Abbreviations

ACTB	Beta-actin
AKT1	AKT serine/threonine kinase 1
AP-1	Activator Protein 1
BC	betweenness centrality
BMA	bone mineralization area
BP	biological processes
CC	cell components
CC	closeness centrality
CCDN1	Cyclin D1
DC	degree centrality
DEX	dexamethasone
DL	drug-likeness
ED	etidronate disodium
EGFR	Epidermal Growth Factor Receptor
ERK 1/2	Extracellular Regulated Protein Kinases
ESR1	Estrogen Receptor 1
FOS	Fos Proto-Oncogene
GO	Gene Ontology
HIF1	Hypoxia Inducible Factor 1
HIF1A	Hypoxia Inducible Factor-1 Subunit Alpha
IL17	Interleukin 17
IL1B	Interleukin 1 Beta
IL6	Interleukin 6
INS	Insulin Resistance
IOD	cumulative optical density
Jun	Transcription factor AP-1
KEGG	Kyoto Encyclopedia of Genes and Genomes
MAPK1	Mitogen-Activated Protein Kinase 1
MAPK3	Mitogen-Activated Protein Kinase 3
MF	molecular functions
NFKB1	Nuclear Factor Kappa B Subunit 1
NF-κB	Nuclear Factor Kappa B Subunit
OB	oral bioavailability
OMIM	Online Mendelian Inheritance in Man
OP	osteoporosis
PBT	polybutylece terephthalate
PI3K	Phosphatidylinositol 3 Kinase
PIK3CA	PI3-Kinase P110 Subunit Alpha
PPI	protein–protein interaction
PTU	1-phenyl-2-thiourea
QCT	quercetin
RelA	Nuclear Factor NF-Kappa-B p65 Subunit
SC	subgraph centrality
TCM	Traditional Chinese Medicine
TCMSP	Traditional Chinese Medicine Systems Pharmacology Database and Analysis Platform
TNF	Tumor Necrosis Factor
TP53	Tumor Protein P53
TTD	Therapeutic Target Database
